# TIL therapy and anti-CTLA4: can they co-exist?

**DOI:** 10.18632/oncotarget.26509

**Published:** 2019-01-01

**Authors:** Marie-Andrée Forget, Cara Haymaker, Rodabe N. Amaria, Chantale Bernatchez

**Affiliations:** Chantale Bernatchez: Department of Melanoma Medical Oncology, The University of Texas MD Anderson Cancer Center (MDACC), Houston, TX, USA; Department of Translational Molecular Pathology, The University of Texas MDACC, Houston, TX, USA; Rodabe N. Amaria: Department of Melanoma Medical Oncology, The University of Texas MD Anderson Cancer Center (MDACC), Houston, TX, USA

**Keywords:** TIL, CTLA4, metastatic melanoma

Adoptive transfer of tumor-infiltrating lymphocytes (TIL ACT) is one of the first living immunotherapies to be tested in multiple clinical trials in metastatic melanoma and results consistently in a 40 to 50% overall response rate [1-5]. The majority of these trials were conducted prior to the wide spread use of checkpoint inhibition. Given the ease of administration and favorable response rates, checkpoint inhibitors such as anti-CTLA4 and anti-PD1 have been FDA approved and are now widely used. This has dramatically changed the patient population seeking TIL therapy. In our recent publication, we evaluated the impact of pretreatment with anti-CTLA4 in TIL ACT-treated patients and found that patients that were exposed to this checkpoint inhibitor prior to TIL therapy experienced a reduced clinical response of shorter duration. In addition, patients who were previously exposed to anti-CTLA4 were infused with less TIL than the checkpoint point naïve patients, suggesting a suboptimal ability of the CTLA4-exposed TIL to expand [6]. Since our previous work had shown that higher number of TIL infused correlated with responses, this observation was of concern [2]. The reduced proliferation occurred only during the rapid expansion protocol (REP) step, where cells are grown in presence of a TCR stimulation. Why would anti-CTLA4 exposed TIL grow less after TCR activation?

**Figure 1:**
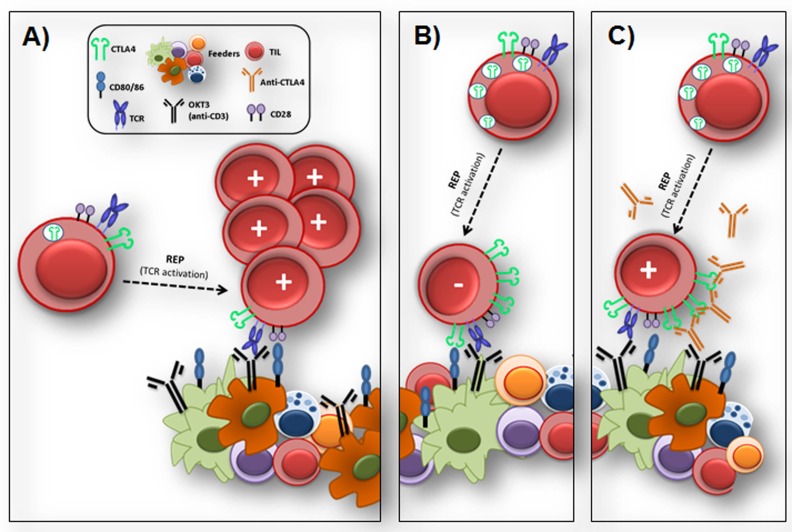
Proposed model for a potential impact of anti-CTLA4 exposure on IL-2 expanded TIL during initial activation in the REP Panel **A.** depicts the expected proliferative outcome after checkpoint naïve IL-2 propagated TIL are activated at the initial step of the REP. TIL are activated through their TCR following interaction with anti-CD3 (OKT3) loaded on the surface of the feeder cells (pool of 3 to 6 normal donors irradiated PBMC). The feeder cells represent an important expansion platform in the initial activation of the TIL, providing TCR stimulation as well as costimulation through expression of CD80/CD86, ligands of the CD28 molecule expressed at the surface of the TIL. Panel **B.** shows the theoretical outcome of a anti-CTLA4 exposed TIL in the initial step of activation of the REP. Unlike the checkpoint naïve TIL, the anti-CTLA4 exposed TIL contain a high level of intracellular CTLA molecules which quickly accumulate at the surface of the TIL at the time of TCR activation through anti-CD3 interaction. Following surface accumulation, the CTLA4 molecules compete against the outnumbered CD28 molecules for binding of the CD80/CD86 molecules. This binding results in suppression of the TIL activation and proliferation, leading to a suboptimal TIL expansion through the REP. Panel **C.** illustrates the protection from suppression provided by the addition of anti-CTLA4 to the REP. Blocking CTLA4 access to CD80/CD86 gives the opportunity for CD28 to interact with the ligands for positive feedback.

A possible explanation was recently brought to our attention by Bjoern et al. Their study elegantly demonstrated that anti-CTLA4 exposed TIL, both CD8 and CD4 subsets, expanded in high dose IL-2 (pre-REP) have increased CTLA4 expression compared to the checkpoint naïve cells [7]. Given that this difference in expression was not on the level of CTLA4 at the surface of the cells but rather on the total CTLA4 (intracellular + extracellular), the presumption is that anti-CTLA4 exposed pre-REP TIL had a higher intracellular pool of CTLA4 molecules. No difference was reported for CD28 expression [7]. These interesting observations led us to hypothesize that the high intracellular pool of CTLA4 may interfere with CD28 costimulation in the REP. Consequently, checkpoint naïve TIL would be provided positive costimulation by interaction of CD28 with the CD80/CD86 molecules following TCR activation by the feeder expansion platform as depicted in panel A of Figure 1. However, as suggested in panel B, the rapid turnover of the CTLA4 molecule at the surface of anti-CTLA4 exposed TIL following TCR activation would create a highly competitive environment against ligation of CD28, benefiting interaction with CTLA4 instead resulting in suppression of the TIL growth. Thus, we postulate that blocking CTLA4 in the REP, concomitantly with TCR activation might provide protection to these pre-exposed TIL and facilitate rapid expansion (Figure 1C). The addition of the anti-CTLA4 antibody at the onset of the REP may correct TIL dysfunction without affecting other cells in the patient since the TIL product is washed several times prior to infusion, avoiding safety concerns.

Although checkpoint blockade has improved survival in a subset of patients, the current clinical challenge is to find therapies which can improve the survival of the 60% of patients who are checkpoint resistant or refractory [8]. There is no clear standard of care therapy for patients with checkpoint inhibitor refractory disease in spite of the intense focus of research in this area in the recent years. Although the TIL regimen presents toxicity concerns, the side effects are predictable, manageable and for the most part reversible. There is no other regimen that has consistently demonstrated better response rates in checkpoint refractory patients. Thus TIL therapy can be a viable approach for the treatment of checkpoint refractory metastatic melanoma patients but more work is needed to generate better cellular products for infusion and to better predict patients most likely to benefit.
